# The miR-125 family is an important regulator of the expression and maintenance of maternal effect genes during preimplantational embryo development

**DOI:** 10.1098/rsob.160181

**Published:** 2016-11-30

**Authors:** Kyeoung-Hwa Kim, You-Mi Seo, Eun-Young Kim, Su-Yeon Lee, Jini Kwon, Jung-Jae Ko, Kyung-Ah Lee

**Affiliations:** 1 Institute of Reproductive Medicine, Department of Biomedical Science, College of Life Science, CHA University, Pangyo, South Korea; 2 Department of Oral Histology-Developmental Biology, School of Dentistry and Dental Research Institute, Seoul National University, Seoul, South Korea

**Keywords:** Sebox, Lin28a, miR-125 family, translational regulation, bioinformatics

## Abstract

Previously, we reported that *Sebox* is a new maternal effect gene (MEG) that is required for early embryo development beyond the two-cell (2C) stage because this gene orchestrates the expression of important genes for zygotic genome activation (ZGA). However, regulators of *Sebox* expression remain unknown. Therefore, the objectives of the present study were to use bioinformatics tools to identify such regulatory microRNAs (miRNAs) and to determine the effects of the identified miRNAs on *Sebox* expression. Using computational algorithms, we identified a motif within the 3′UTR of *Sebox* mRNA that is specific to the seed region of the miR-125 family, which includes miR-125a-5p, miR-125b-5p and miR-351-5p. During our search for miRNAs, we found that the *Lin28a* 3′UTR also contains the same binding motif for the seed region of the miR-125 family. In addition, we confirmed that Lin28a also plays a role as a MEG and affects ZGA at the 2C stage, without affecting oocyte maturation or fertilization. Thus, we provide the first report indicating that the miR-125 family plays a crucial role in regulating MEGs related to the 2C block and in regulating ZGA through methods such as affecting Sebox and Lin28a in oocytes and embryos.

## 1. Background

Gene expression is a multi-step process that is regulated at both the transcriptional and translational levels as well as by the turnover of mRNAs and proteins [1]. MicroRNAs (miRNAs) are a class of endogenous, single-stranded and non-coding small RNAs (approximately 21–25 nucleotides) that primarily bind to complementary sequences in the 3′UTRs of their target mRNAs; this binding results in mRNA degradation and/or repression of mRNA translation [2]. Complete complementarity between miRNA and mRNA rarely occurs in mammals, but binding at the seed region (6–8 nucleotides at the 5′ end of the miRNA that exactly complements the target mRNA) sufficiently suppresses expression of that specific gene [3]. In general, one gene can be regulated by several miRNAs, and one miRNA can regulate the expression of several target genes [3].

miRNAs are known to play an essential role in the regulation of normal development, disease status and many other physiological processes, including fertility. miRNAs exhibit dynamic expression profiles in oocytes and regulate the expression of many maternal genes in oocytes and embryos. Specifically, disruption of *Dicer*, a key enzyme involved in miRNA processing, results in meiotic arrest and severe spindle and chromosomal segregation defects in oocytes [4]. Some miRNAs are abundant in the immature oocyte and are then depleted throughout oocyte maturation, while others are relatively stable [5]. The differential expression of miRNAs is spatially and temporally regulated during the completion of oocyte meiosis and early embryo development [6]. Interestingly, the clustered miRNAs miR-430 and miR-309 have been linked to maternal mRNA clearance during the maternal-to-zygotic transition (MZT) [7,8].

During oogenesis, maternal effect genes (MEGs) are produced and accumulate in oocytes and function in the completion of fertilization, embryonic cell division, zygotic genome activation (ZGA) and early embryogenesis [9]. Abnormalities in the expression of MEGs result in defective embryogenesis [10], and disruption of MEGs such as *Mater* [11], *Ube2a* [12], *Brg1* [13], *Padi6* [14], *Basonuclin* [15] and *Nlrp2* [16] disrupts ZGA and impairs embryo development, causing arrest at the two-cell (2C) stage, referred to as the 2C block in mice.

In a previous study, we found that the skin-embryo-brain-oocyte homeobox (*Sebox*) is also required for normal early embryo development, especially development at the 2C stage, and reported *Sebox* as a new candidate MEG [17]. *Sebox* is a mouse paired-like homeobox gene that encodes a homeodomain-containing protein [18]. Compared with its expression in metaphase II (MII) oocytes, *Sebox* expression is high in germinal vesicle (GV) oocytes and persists until ZGA occurs in mice [17]. Despite the specific and marked silencing of *Sebox* through *Sebox* RNA interference (RNAi), the oocytes' maturation rate, spindle configuration, chromosome organization and gross morphology were not affected [17]. However, silencing *Sebox* at the pronuclear (PN) stage arrested embryonic development at the 2C stage [17]. We confirmed that this developmental arrest in *Sebox*-silenced embryos was due to the incomplete degradation of several maternal factors (*c-mos*, *Gbx2* and *Gdf9*), with the concurrent aberrant expression of certain ZGA markers (*Mt1a*, *Rpl23*, *Ube2a* and *Wee1*) [19]. Despite the importance of Sebox in the regulation of embryo development, regulatory mechanisms for *Sebox* expression are not yet well understood.

Therefore, the objectives of the present study were to identify miRNAs that regulate *Sebox* expression and to evaluate their function during preimplantational embryonic development. While we were cross-checking multiple computational algorithms, we discovered that the seed region of miR-125 family members is specific to the miRNA response element (MRE) within the 3′UTR of *Sebox* mRNA. In addition, these computational methods also revealed that Lin-28 homologue A (*Lin28a*) mRNA has the same conserved miR-125 family target site in its 3′UTR region. Therefore, we extended the aim of our study to include evaluation of the regulatory effect of the miR-125 family on the expression of *Lin28a* as well as *Sebox*.

## 2. Material and methods

### 2.1. Animals

Female imprinting control region (ICR) mice were obtained from Koatech (Pyeoungtack) and mated to male mice of the same strain to produce embryos in the breeding facility at the CHA Stem Cell Institute of CHA University. All described procedures were reviewed and approved by the University of Science Institutional Animal Care and Use Committee and were conducted in accordance with the Guiding Principles for the Care and Use of Laboratory Animals.

### 2.2. Reagents

Chemicals and reagents were obtained from Sigma Chemical Corporation unless otherwise noted.

### 2.3. Isolation of oocytes and embryos

To isolate GV oocytes from preovulatory follicles, four-week-old female ICR mice were injected with 5 IU of eCG and sacrificed 46 h later. Cumulus-enclosed oocyte complexes (COCs) were recovered from the ovaries by puncturing the preovulatory follicles with a 27-gauge needle. M2 medium containing 0.2 mM 3-isobutyl-1-methyl-xanthine (IBMX) was used to inhibit germinal vesicle breakdown (GVBD). Isolated oocytes were snap-frozen and stored at −70°C prior to RNA isolation. To obtain MII oocytes, we injected female mice with 5 IU eCG, followed by 5 IU hCG after 46 h. Superovulated MII oocytes were obtained from the oviduct 16 h after hCG injection. Cumulus cells surrounding MII oocytes were removed by treating COCs with hyaluronidase (300 U ml^−1^). Female mice in which superovulation was induced were mated, and embryos were obtained at specific time points after hCG injection as follows: PN 1-cell embryo at 18–20 h, 2C embryos at 44–46 h, 4C embryos at 56–58 h, 8C embryos at 68–70 h, morula (MO) stage at 80–85 h and blastocyst (BL) stage at 96–98 h.

### 2.4. Microinjection and *in vitro* culture

The GV oocytes were microinjected with miRNA mimics in M2 medium containing 0.2 mM IBMX. An injection pipette containing the miRNA mimic solution was inserted into the cytoplasm of an oocyte, and 10 pl of 2 μM miRNA mimic, 2 μM miRNA inhibitor or *Lin28a* small interfering RNA (siRNA; Dharmacon) was microinjected using a constant flow system (Femtojet; Eppendorf). To assess injection damage, oocytes were injected with a negative control miRNA mimic and control siRNA (Dharmacon), which were used as negative controls. To determine the rate of *in vitro* maturation, oocytes were cultured in M16 medium containing 0.2 mM IBMX for 24 h and then cultured in M16 medium alone for 16 h in 5% CO_2_ at 37°C. After the miRNA mimic microinjection experiments, the maturation stage of the oocytes was scored based on the presence of a GV oocyte, a polar body (MII oocyte) or neither a GV nor a polar body (MI oocyte).

### 2.5. Messenger RNA isolation in oocytes and quantitative real-time RT-PCR

Oocyte mRNA was isolated using the Dynabeads mRNA DIRECT Kit (Invitrogen Dynal AS) according to the manufacturer's instructions. Briefly, oocytes were suspended with lysis/binding buffer and mixed with pre-washed Dynabeads oligo dT_25_. After RNA binding, the beads were washed twice with buffer A and then with buffer B, and RNA was eluted with Tris–HCl via incubation at 72°C. The isolated mRNA was used as a template for reverse transcription using oligo(dT) primers according to the M-MLV protocol. PCR was performed with a single-oocyte-equivalent amount of cDNAs. The PCR conditions and primer sequences for the encoding genes are listed in table 1. Gene expression was quantified via real-time RT-PCR, as described previously [17].

**Table 1.** Primer sequences and RT-PCR conditions. The annealing temperature was 60°C for all genes. F, forward primer; R, reverse primer.

gene	primer sequence	product size
*Lin28a*	F: 5'-GCGAAGATCCAAAGGAGACA-3'	206 bp
	R: 5'-TGTGGATCTCTTCCTCTTCC-3'	
*Oct4*	F: 5'-CCGGAAGAGAAAGCGAACTA-3'	393 bp
	R: 5'-CAGTTTGAATGCATGGGAGA-3'	
*Klf4*	F: 5'-AAAAGAACAGCCACCCACAC-3'	227 bp
	R: 5'-GAAAAGGCCCTGTCACACTT-3'	
*Sox2*	F: 5'-AACCCCAAGATGCACAACTC-3'	201 bp
	R: 5'-TCCGGGAAGCGTGTACTTAT-3'	
*c-Myc*	F: 5'-TGATGTGGTGTCTGTGGAGA-3'	230 bp
	R: 5'-TGTTGCTGATCTGCTTCAGG-3'	
*Nanog*	F: 5'-CCAAAGGATGAAGTGCAAGC-3'	106 bp
	R: 5'-GCAATGGATGCTGGGATACT-3'	
*Dppa2*	F: 5'-CACAGACTACGCTACGCAATCA-3'	245 bp
	R: 5'-AGTGTCTCCGAAGTCTCAAATAG-3'	
*Dppa4*	F: 5'-GATACCTGCCCTCATTGACCCT-3'	182 bp
	R: 5'-CACACCACATTTCCCCTTTGACTTC-3'	
*Gata6*	F: 5'-CAACCACTACCTTATGGCGTAGAAA-3'	354 bp
	R: 5'-GGCCGTCTTGACCTGAATACTTGA-3'	
*Piwil2*	F: 5'-TTGTCATGTCGGACGGGAAGG-3'	320 bp
	R: 5'-CTCATTGCTGGCTGTCTCGTTTTGT-3'	
*Cbx1*	F: 5'-CTACGAGCAGTGTCACCCTTCA-3'	295 bp
	R: 5'-TTGCCTCCCTCTGACTTATCTG-3'	
*Hdac3*	F: 5'-TCCCGAGGAGAACTACAGCAGG-3'	280 bp
	R: 5'-GGACAATCATCAGGCCGTGAGA-3'	
*Tbpl1*	F: 5'-CGGAACAACAAAGCGAGAAACC-3'	203 bp
	R: 5'-AGATCACATCCGTGGGAAGACG-3'	
*eIF-1a*	F: 5'-TTTGGTCACTACTCAGGAGG-3'	149 bp
	R: 5'-ATCAGAAGCAACTGGGACAC-3'	
*Mt1a*	F: 5'-CACCAGATCTCGGAATGGAC-3'	114 bp
	R: 5'-AGCAGCTCTTCTTGCAGGAG-3'	
*Cdc2*	F: 5′-GGACTACAAGAACACCTTTC-3′	262 bp
	R: 5′-CAGGAAGAGAGCCAACGGTA-3′	
*Hsp70.1*	F: 5′-AACGTGCTCATCTTCGACCT-3′	185 bp
	R: 5′-TGGCTGATGTCCTTCTTGTG-3′	
*Muerv-l*	F: 5′-TTGCTTCCTGTCCCCATAAC-3′	132 bp
	R: 5′-AAAATGACCAGGGGGAAGTC-3′	
*Rpl23*	F: 5′-CATGGTGATGGCCACAGTTA-3′	136 bp
	R: 5′-GACCCCTGCGTTATCTTCAA-3′	
*Ube2a*	F: 5′-AATGGTTTGGAATGCGGTCA-3′	272 bp
	R: 5′-TGTTTGCTGGACTATTGGGA-3′	
*U2afbp-rs*	F: 5′-TAAGCTGCAACCTGGAACCT-3′	109 bp
	R: 5′-CCTGCGTACCATCTTCCATT-3′	
*H1Foo*	F: 5'-GCGAAACCGAAAGAGGTCAGAA-3'	377 bp
	R: 5'-TGGAGGAGGTCTTGGGAAGTAA-3'	
*Gapdh*	F: 5'-ACCACAGTCCATGCCATCAC-3'	452 bp
	R: 5'-TCCACCACCCTGTTGCTGTA-3'	

### 2.6. Isolation of oocyte mature miRNA and quantitative real-time RT-PCR for miRNA

Mature miRNA was isolated from 20 GV and MII oocytes using a miRNeasy micro kit (Qiagen) and bacterial ribosomal RNA carrier (Roche) and reverse-transcribed with miScript II RT (Qiagen). To quantify the mRNA, quantitative real-time RT-PCR was performed with a single-oocyte-equivalent amount of cDNA as previously described [20]. The PCR conditions and primer sequences are shown in table 1. The quantitative real-time RT-PCR analysis was performed with a miScript SYBR Green PCR kit (Qiagen) and iCycler (Bio-Rad) with universal tag primers and specific mature miRNA primers (miScript Primer Assay; Qiagen). The expression level of each miRNA species in oocytes was normalized to that of RUN6-2. The relative expression levels of the target miRNAs were calculated using the 2^−ΔΔCt^ method, and all analytic procedures were repeated at least three times.

### 2.7. Parthenogenetic activation and culture of activated oocytes

Mature oocytes were parthenogenetically activated by culturing them for 2 h in Ca^2^-free KSOM medium supplemented with 10 mM SrCl_2_ and 5 mg ml^−1^ cytochalasin B. The activated oocytes were then cultured in modified Chatot, Ziomek and Bavister (CZB) medium (37°C, 5% CO_2_) to monitor their development to the 2C stage.

### 2.8. *In vitro* fertilization

Sperm were collected from the caudal epididymides of eight-week-old male ICR mice (Koatech). The epididymis was incised to release sperm into M16 medium. The sperm were incubated in M16 medium for 1 h to allow capacitation. The zona pellucida (ZP) was removed from oocytes by treating them with Tyrode's solution (pH 2.5). After ZP thinning was observed using a microscope, the oocytes were transferred to M16 medium, and the cellular mass was washed out of the ZP by gentle pipetting. ZP-free MII eggs were then placed in a 200 μl droplet of M16 medium under mineral oil and inseminated with 2.5 × 10^4^ ml^−1^ sperm. After 2 h, the oocytes were washed to remove unbound sperm and cultured in M16 medium for 5 h (37°C, 5% CO_2_) to observe PN formation.

### 2.9. Transcriptional activity assay

Newly synthesized RNAs (indicative of transcriptional activity) were visualized in embryos by applying 5-ethynyl uridine (EU) in an *in vitro* embryonic transcriptional activity assay [19,21]. The Click-iT RNA Imaging Kit (Thermo Fisher Scientific) was used for these assays. After subjecting embryos to culture in 2 mM EU-supplemented M16 medium, the embryos were washed and fixed in 3.7% formaldehyde containing 0.2% Triton X-100. Finally, the embryos were sequentially immersed in reaction buffer, washed three times and examined via confocal microscopy (Leica).

### 2.10. Cell culture and miRNA mimic transfection

J1 mouse embryonic stem cells (mESCs) were purchased from ATCC. The mESCs were cultured in Dulbecco's modified Eagle's medium supplemented with 15% fetal calf serum (HyClone), 0.1 mM 2-mercaptoethanol (Sigma), 100 U ml^−1^ penicillin, 100 μg ml^−1^ streptomycin, 2 mM glutamine (Gibco) and 1000 U ml^−1^ LIF (Chemicon). The cells were routinely passaged at 80–90% confluence. The mESCs were transfected with 10 nM of each miRNA mimic (negative control, miR-125a-5p mimic, miR-125b-5p mimic and miR-351-5p mimic; Bioneer) using Lipofectamine 2000 (Invitrogen Corporation) transfection reagent in Opti-MEM media (Invitrogen Corporation) according to the manufacturer's directions. After 48 h, the cells were harvested for gene expression analysis.

### 2.11. Western blotting

Protein extracts were separated using 12% SDS-PAGE and transferred onto polyvinylidene difluoride membranes (Amersham Biosciences). The membranes were blocked for 1 h in Tris-buffered saline-Tween (TBST; 0.2 M NaCl, 0.1% Tween-20 and 10 mM Tris (pH 7.4)) containing 5% non-fat dry milk. The blocked membranes were then incubated with a goat polyclonal anti-Sebox antibody [17], goat polyclonal anti-c-Myc antibody (1 : 1000; Abcam), rabbit polyclonal anti-Lin28a antibody (1 : 1000; Abcam) or mouse monoclonal anti-α-tubulin antibody (1 : 1000; sc-8035, Santa Cruz Biotechnology) in TBST. After the incubation, the membranes were incubated with horseradish peroxidase-conjugated anti-goat IgG (1 : 2000; A5420), anti-rabbit IgG (1 : 2000) or anti-mouse IgG (1 : 2000; A-2554) in TBST for 1 h at room temperature. After each step, the membranes were washed several times with TBST, and bound antibody was detected using an enhanced chemiluminescence detection system (Santa Cruz Biotechnology) according to the manufacturer's instructions.

### 2.12. DNA constructs

To generate the *Sebox* firefly luciferase reporter, the full-length sequence of the *Sebox* 3′UTR (NM_008759.2: 611-1285), encompassing the MRE of miR-125a-5p, miR-125b-5p and miR-351-5p, was amplified using the forward primer GCTAGCTTTAGGTGTAGAGCTTTTAAGT and the reverse primer AAGCTTTTAAAGCAAAGAGTTTTGTTTT. To generate the *Lin28a* firefly luciferase reporter, the sequence of the *Lin28a* 3′UTR (NM_145833.1: 1193-1846)-containing MRE was amplified using the forward primer GCTAGCGATGACAGGCAAAGAGGGTG and the reverse primer AAGCTTAGGCTTCCACTAATCTGGCA. The *Sebox* 3′UTR (675 bp) and *Lin28a* 3′UTR (654 bp) PCR products were digested with *Nhe*I and *Hind*III. Then, the digested PCR products were cloned into a *Nhe*I- and *Hind*III-opened pGL4.10 vector (Promega). The sequences of the constructs were confirmed by DNA sequencing.

### 2.13. Luciferase reporter assay

The effect of miRNA overexpression on the mouse *Sebox* or *Lin28a* 3′UTR was assessed in HEK 293 cells. Cells grown in 24-well plates were transfected with the following plasmids using Lipofectamine 2000 (Invitrogen Corporation): 100 ng of a plasmid encoding a firefly luciferase reporter fused with the mouse *Sebox* or *Lin28a* 3′UTR, 50 ng pRL-TK control plasmid (*Renilla* luciferase) and 10 nM miRNA mimics with or without 50 nM inhibitors. The cells were harvested 48 h later and assayed with a dual-luciferase assay system (Promega) and a Modulus microplate reader luminometer (Turner Biosystems). For signal normalization, the readout of firefly luciferase activity from non-transfected HEK 293 cells was subtracted as background, and the activity of the firefly luciferase was then normalized to that of the co-transfected control *Renilla* luciferase. All results are the mean of at least three independent experiments. Significant downregulation of normalized luciferase expression was identified using paired *t*-tests.

### 2.14. Statistical analysis

The quantitative real-time RT-PCR data were statistically analysed using Student's *t*-tests. The data derived from at least three separate and independent experiments were expressed as the mean ± s.e.m. The *p*-values for each gene in the miRNA mimic or miRNA inhibitor microinjection group and the negative control miRNA microinjection group were calculated based on paired *t-*tests of triplicate ΔC_T_ values, and a value of *p* < 0.05 was considered statistically significant.

## 3. Results

### 3.1. The miR-125 family is predicted to target *Sebox* as well as *Lin28a*

**Figure 1.**
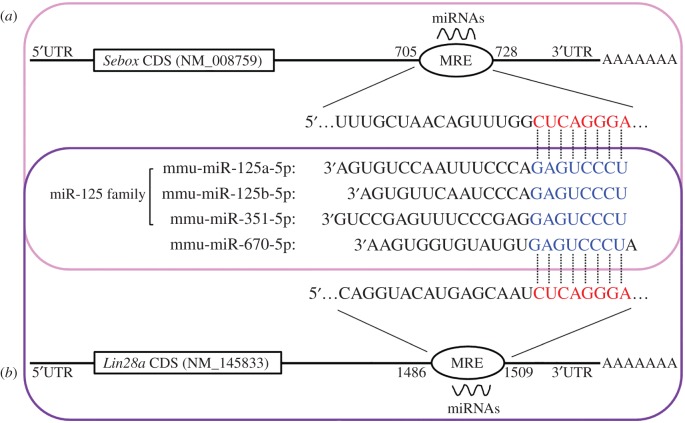
miRNAs predicted to target *Sebox* and *Lin28a*. The predicted miRNA (miR-125a-5p, miR-125b-5p, miR-351-5p and miR-670-5p) targeting sequence located in the 3′UTR of *Sebox* (*a*, pink box) and/or *Lin28a* (*b*, violet box) mRNA was found using TargetScan, miRanda and miRmap. Red letters indicate the MRE in the 3′UTR of *Sebox* and *Lin28a* mRNA, while blue letters indicate the seed region of each miRNA we identified.

Computational approaches have become an indispensable tool for understanding and predicting miRNA target gene interactions. To identify miRNAs capable of targeting *Sebox*, we identified potential miRNA binding sites, known as MREs, in the *Sebox* 3′UTR using bioinformatic tools and then confirmed whether those putative sites were indeed functional. Because miRNAs bind to their targets with imperfect complementary sequences, algorithms that predict miRNA binding sites in single mRNAs often produce a large number of false positives. We used TargetScan (http://www.targetscan.org), miRanda (http://www.microrna.org) and miRmap (http://mirmap.ezlab.org) to select sequences showing complete complementarity between the miRNA seed region and the 3′UTR of the mRNA; these algorithm-based tools help to eliminate false positives. We identified one predicted MRE in the 3′UTR of *Sebox* mRNA (NM_008759: position 721–728) and four miRNAs (miR-125a-5p, miR-125b-5p, miR-351-5p and miR-670-5p) that were predicted to target *Sebox* (figure 1*a*, pink box). Among these four putative miRNAs identified using TargetScan, miRanda and miRmap, miR-125a-5p, miR-125b-5p and miR-351-5p are members of the miR-125 family, whereas miR-670-5p is not (figure 1). Interestingly, the same sequence of the MRE predicted for the four miRNAs was also found in the 3′UTR of *Lin28a* mRNA (NM_145833: position 1502–1509; figure 1*b*, violet box). Each computational algorithm provides a score that can be employed to predict mRNA–miRNA interactions. These algorithms use different strategies to rank predictions; in some cases, the larger positive values represent more trustable predictions, whereas in other cases, the smaller values are better. Using these three algorithms, we identified a target prediction score for the interactions between each miR-125 family member and the target mRNAs, *Sebox* and *Lin28a*. The electronic supplementary material, table S1 lists the estimated prediction score for the interactions between the miR-125 family members and each target MEG. We found that the sequences with higher scoring predictions were the strongest candidates for validation experiments, and we identified miR-125 family binding sites in the *Sebox* and *Lin28a* mRNAs (electronic supplementary material, table S1).

### 3.2. miR-125 family members are expressed in oocytes and embryos

**Figure 2.**
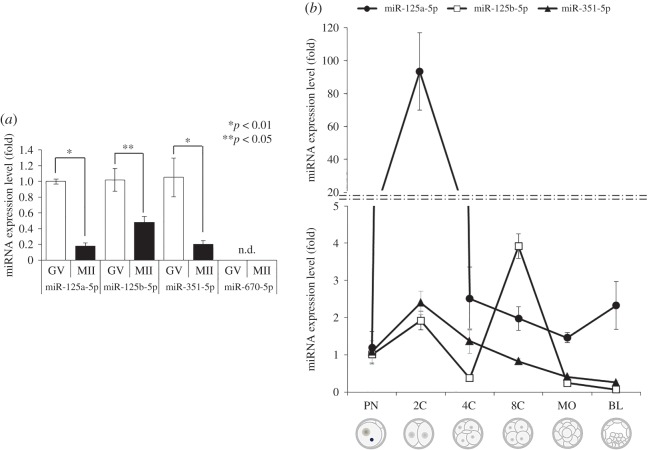
Expression of miR-125 family members in oocytes and early developmental embryos. (*a*) Expression of miR-125 family members was detected in oocytes. Expression of the four predicted miRNAs in mouse GV and MII oocytes was evaluated using quantitative real-time RT-PCR analysis with mature miRNA primers. n.d., detected. *p < 0.01, **p < 0.05, relative to the expression level in GV oocytes. (*b*) Relative expression levels of miR-125 family members during early embryogenesis. The relative expression of miR-125a-5p, miR-125b-5p and miR-351-5p in single embryos throughout development was measured by quantitative real-time RT-PCR. Experiments were repeated at least three times, and the data are expressed as the mean ± s.e.m. Filled circles, miR-125a-5p; open squares, miR-125b-5p; filled triangles, miR-351-5p.

Expression profiles of the members of the miR-125 family were examined in oocytes and early developmental stage embryos via quantitative real-time RT-PCR. We found that the members of the miR-125 family were expressed in oocytes, whereas miR-670-5p was not (figure 2*a*). Expression of the miR-125 family members was higher in GV oocytes than in MII oocytes (figure 2*a*). Because miR-670-5p was not detected in oocytes, we decided to exclude miR-670-5p from further investigation. miR-125a-5p expression was detected in PN-stage embryos, dramatically increased at the 2C stage, and then decreased at stage 4C and after (figure 2*b*; filled circles). miR-125b-5p expression was detected in PN-stage embryos, slightly increased at the 2C stage, and then decreased at the 4C stage. Interestingly, miR-125b-5p expression dramatically increased at the 8C stage and then decreased to an undetectable level from MO- to BL-stage embryos (figure 2*b*; open squares). miR-351-5p expression was detected in PN-stage embryos, slightly increased at the 2C stage and gradually decreased thereafter (figure 2*b*; filled triangles).

### 3.3. The miR-125 family directly targets the 3′UTR of *Sebox* and *Lin28a*

The *in silico* analysis of putative miRNAs and their targets *Sebox* and *Lin28a* indicated that miR-125a-5p, miR-125b-5p and miR-351-5p may regulate the expression of *Sebox* and *Lin28a*. Therefore, we sought to determine whether the miR-125 family can regulate *Sebox* and *Lin28a* expression in oocytes and undifferentiated mESCs. As an assay of miRNA function, we evaluated the ability of each miR-125 family member to target endogenous *Sebox* and *Lin28a* mRNAs in oocytes and mESCs by microinjecting or transfecting cells with mimics of each miR-125 family member. When oocytes were microinjected with a mimic of miR-125a-5p or miR-125b-5p, *Sebox* transcript levels were markedly reduced (electronic supplementary material, figure S1*a*). Unexpectedly, microinjection of the miR-351-5p mimic significantly increased oocyte-stored *Sebox* mRNA (electronic supplementary material, figure S1*a*). Several studies have reported that the endogenous miRNA targets have significantly higher expression levels following the transfection of mimics due to the low effectiveness of suppression of the endogenous miRNA pathway [15,22]. *Sebox* mRNA levels were slightly increased when mESCs were transfected with miR-125a-5p or miR-125b-5p mimics, whereas *Sebox* mRNA levels were slightly decreased following miR-351-5p mimic transfection, but none of these changes were statistically significant (electronic supplementary material, figure S1*b*). These results show that treating oocytes with mimics of miR-125 family members resulted in an inconsistent pattern of endogenous *Sebox* mRNA regulation. However, the expression of *Lin28a* transcripts in oocytes was markedly reduced when the oocytes were microinjected with mimics of each miR-125 family member (electronic supplementary material, figure S1*c*). *Lin28a* mRNA levels were significantly decreased when mESCs were transfected with miR-125a-5p and miR-125b-5p but not when they were transfected with miR-351-5p (electronic supplementary material, figure S1*d*). These results indicate that the identified miRNAs did regulate the expression of the target genes *Sebox* and *Lin28a*, but the transcript expression profiles of *Sebox* and *Lin28a* varied among the different cell types.

### 3.4. Mimics and inhibitors of the miR-125 family regulate the level of Sebox and Lin28a translation

**Figure 3.**
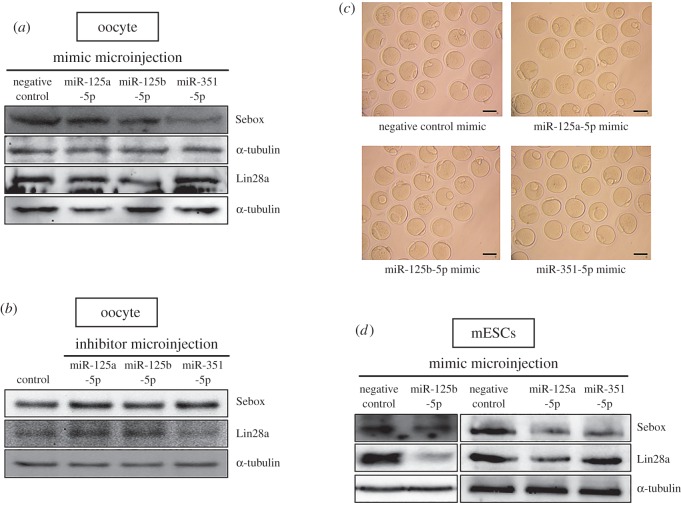
The miR-125 family regulates Sebox and Lin28a translation. Endogenous Sebox and Lin28a protein expression levels were detected by western blotting after miR-125 family members were overexpressed (*a*) or downregulated (*b*) in oocytes. The blots were reprobed with α-tubulin antibody as a loading control. (*c*) Photomicrographs of MII oocytes cultured *in vitro* after microinjection with mimics of each miR-125 family member. Disruption of Sebox and Lin28a translation by microinjection of mimics of each miR-125 family member did not affect normal oocyte nuclear maturation. Scale bars, 100 µm. (*d*) Western blotting was performed to detect the endogenous expression of Sebox and Lin28a in mESCs transfected with mimics of miR-125 family members.

We analysed whether overexpression of miR-125 family members affected Sebox and Lin28a protein expression. Sebox translational levels were negatively regulated by the overexpression of mimics of each miR-125 family member in oocytes (figure 3*a*), while enhanced Sebox protein expression was observed in oocytes that were microinjected with inhibitors of miR-125 family members (figure 3*b*). In addition, Lin28a protein translation was also reduced when oocytes were microinjected with mimics of miR-125a-5p and miR-125b-5p but not when they were microinjected with miR-351-5p mimics (figure 3*a*). However, after the levels of miR-125a-5p and miR-125b-5p were reduced in oocytes via inhibitor microinjection, Lin28a protein levels were also markedly elevated (figure 3*b*). In this case, the inhibitor of miR-351-5p again led to different results compared with the inhibitors of miR-125a-5p and miR-125b-5p (figure 3*b*). Although Sebox and Lin28a protein expression was markedly reduced following the microinjection of mimics of each miR-125 family member (figure 3*a*), oocyte nuclear maturation was not affected, and most of the oocytes extruded polar bodies and matured to the MII stage (figure 3*c* and table 2). No change in maturation rate was observed in the miR-125a-5p (91.3 ± 2.4), miR-125b-5p (92.3 ± 5.0), miR-351-5p (95.3 ± 3.0) or negative control (90.2 ± 3.2; figure 3*c* and table 2) groups. These results are highly supportive of our earlier data, indicating that the reduction of Sebox and Lin28a expression by the miR-125 family members did not affect the regulation of oocyte nuclear maturation.

**Table 2.** *In vitro* maturation of mouse oocytes after GV oocytes were injected with mimics of each miR-125 family member. Common letters indicate no significant difference.

	number of oocytes (%)			
	total	metaphase I	metaphase II	abnormal
negative control mimic	133	12 (9.0)^a^	120 (90.2)^b^	1 (0.8)^c^
miR-125a-5p mimic	127	11 (8.7)^a^	116 (91.3)^b^	0 (0.0)^c^
miR-125b-5p mimic	155	10 (6.5)^a^	143 (92.3)^b^	2 (1.3)^c^
miR-351-5p mimic	149	5 (3.4)^a^	142 (95.3)^b^	2 (1.3)^c^

After miR-125 family members were overexpressed in mESCs, Sebox protein levels were decreased (figure 3*d*). In addition, Lin28a protein levels were also markedly decreased in mESCs in which miR-125a-5p or miR-125b-5p was overexpressed (figure 3*d*). However, miR-351-5p overexpression did not affect Lin28a translation levels in mESCs (figure 3*d*). We found that miR-125a-5p and miR-125b-5p inhibited both Sebox and Lin28a protein expression, whereas miR-351-5p suppressed only Sebox protein expression.

**Figure 4.**
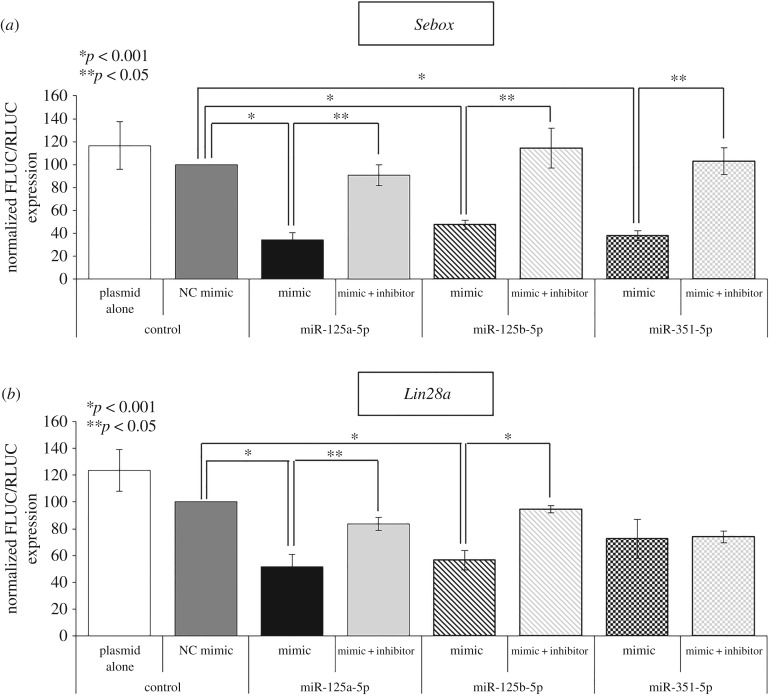
Sebox and Lin28a are direct downstream targets of the miR-125 family. HEK 293T cell reporter-based assays of miR-125a-5p, miR-125b-5p and miR-351-5p targeting of *Sebox* mRNA (*a*) and *Lin28a* mRNA (*b*) through a targeting sequence located at the 3′UTR. Luciferase assays were performed to determine the effects of miRNA overexpression with or without inhibition of the *Sebox* or *Lin28a* 3′UTR. The level of firefly luciferase (FLUC) activity, normalized against that of *Renilla* luciferase (RLUC), is presented as a percentage of the signal obtained with transfection of a negative control (NC) mimic. Experiments were repeated at least three times, and the data are expressed as the mean ± s.e.m. *Renilla* luciferase was commonly used as an internal control. Plasmid alone and NC mimic groups were used as controls. *p < 0.001, **p < 0.05 compared with the NC mimic or with the mimic of each miR-125 family member.

To confirm that the direct pairing of miR-125 family members and their target mRNA leads to suppression of translation, we performed a luciferase reporter assay. We measured the ability of miRNA overexpression to decrease the expression of firefly luciferase from an mRNA bearing the 3′UTR of *Sebox* (figure 4*a*) and *Lin28a* (figure 4*b*). We found that overexpression of all of the miRNAs significantly reduced the expression of firefly luciferase via targeting of the mouse *Sebox* 3′UTR. When miRNA mimics of miR-125a-5p (black bar), miR-125b-5p (black striped bar) and miR-351-5p (black checked bar) were each co-transfected with the *Sebox* 3′UTR luciferase reporter, the expression of firefly luciferase was decreased (by 2.9-fold, 2.1-fold and 2.6-fold, respectively) relative to the luciferase expression observed following transfection with the negative control mimic (dark grey bar; figure 4*a*). This reduction in expression by members of the miR-125 family was blocked by treatment with an inhibitor of each miRNA (figure 4*a*; light grey bar, light grey striped bar and light grey checked bar). Consistent with the decreased endogenous expression of *Sebox*, the luciferase activity for *Lin28a* expression was also significantly decreased when miR-125a-5p (black bar) or miR-125b-3p (black striped bar) was transfected (figure 4*b*). Meanwhile, miR-351-5p (black checked bar) had no effect on *Lin28a* expression (figure 4*b*), which is also consistent with the western blotting results (figure 3). These results indicate that direct binding between the miR-125 family members and the MRE in the 3′UTR of *Sebox* or *Lin28a* mRNA is capable of inhibiting *Sebox* and *Lin28a* post-transcriptional expression.

### 3.5. *Lin28a* is a new maternal effect gene

Using computational approaches, the putative MRE of the miR-125 family was identified in the 3′UTR of *Sebox* and *Lin28a* (figure 1). Lin28a regulates the translation of genes important for ESC growth and maintenance; however, its function in mammalian oocytes and early developmental stage embryos is not known. Therefore, we investigated the potential role of *Lin28a* in oocyte maturation, fertilization and preimplantation embryonic development and determined whether *Lin28a* functions as a MEG like *Sebox*.

#### 3.5.1. *In vitro* oocyte maturation occurs despite *Lin28a* RNAi

Like the levels of many other maternally expressed mRNAs, *Lin28a* mRNA levels were higher in oocytes than in embryos (electronic supplementary material, figure S2*a*) and were particularly high in GV oocytes (electronic supplementary material, figure S2*b*). Lin28a protein levels were also decreased during oocyte maturation (electronic supplementary material, figure S2*c*). To determine the role of Lin28a during oocyte maturation, *Lin28a* siRNA was microinjected into the cytoplasm of GV oocytes to silence Lin28a expression. Despite the knockdown of *Lin28a* mRNA (electronic supplementary material, figure S2*d*) and protein expression (electronic supplementary material, figure S2*e*), the oocytes all matured, and the MII oocytes were morphologically normal (table 3), suggesting that Lin28a is not a critical factor for nuclear maturation in mouse oocytes. A recent study reported that Lin28a regulates Oct4 at the post-transcriptional stage in human ESCs [23]. Oct4 binds to its own promoter region, Sox2 and Nanog also bind to the *Oct4* promoter and Nanog binds to the promoters of all three factors. Thus, we evaluated the expression of other reprogramming factors in oocytes after the injection of *Lin28a* siRNA and found that *c-Myc* mRNA and protein levels in MII oocytes were considerably reduced by *Lin28a* siRNA (electronic supplementary material, figure S2*d*,*e*). However, other reprogramming factors (*Oct4*, *Klf4* and *Sox2*) did not appear to be affected (electronic supplementary material, figure S2*d*).

**Table 3.** *In vitro* maturation of mouse GV oocytes after *Lin28a* siRNA injection. Common letters indicate no significant difference.

treatment	number of oocytes (%)			
	total	GV	metaphase I	metaphase II
no injection	190	3 (1.6)^a^	19 (10.0)^b^	168 (88.4)^c^
control siRNA injection	142	1 (0.7)^a^	25 (17.6)^b^	116 (81.4)^c^
*Lin28a* siRNA injection	168	0 (0.0)^a^	12 (7.1)^b^	156 (92.9)^c^

**Figure 5.**
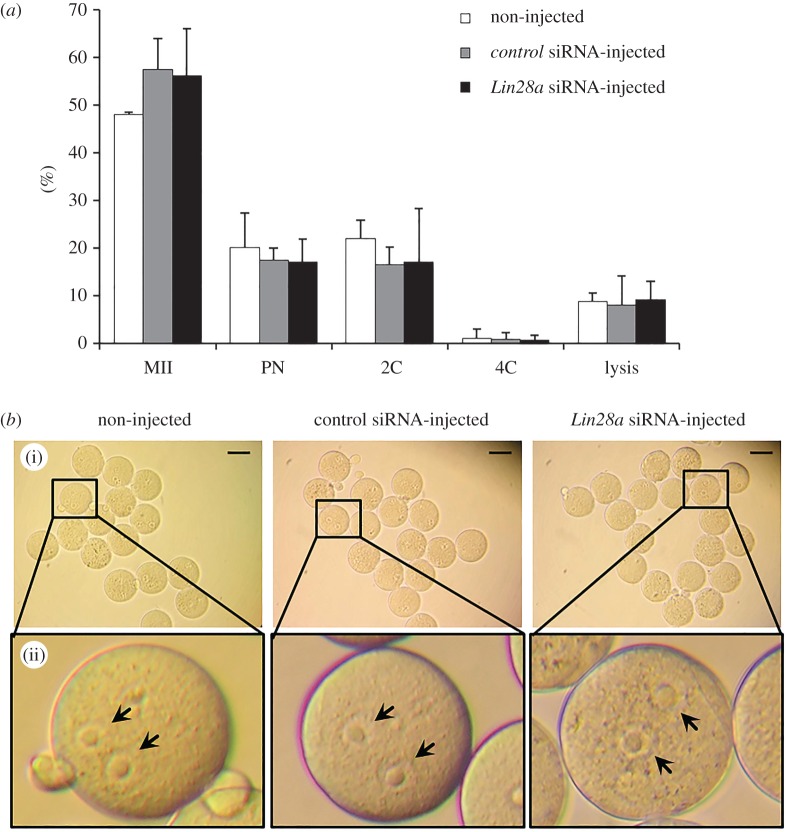
*Lin28a*-silenced MII oocytes were activated parthenogenetically and formed PN embryos after fertilization. (*a*) GV oocytes injected with control siRNA or *Lin28a* siRNA were matured *in vitro* for 14 h. MII oocytes were activated with SrCl_2_ and cultured in CZB medium to observe parthenogenetic development. Activated oocytes were scored according to developmental stage. The results are expressed as the mean ± s.e.m. of three independent experiments. (*b*) Despite the reduction of Lin28a in MII oocytes, sperm penetration and PN formation occurred. Photomicrographs of PN-stage embryos after IVF. MII oocytes (i) injected with control siRNA or *Lin28a* siRNA appear similar to control oocytes. The images in (ii) are high-magnification images of the boxed area in (i). Arrows indicate well-formed pronuclei in the oocyte cytoplasm. Scale bars, 100 µm.

To verify that complete oocyte cytoplasmic maturation occurred after RNAi, *Lin28a*-silenced MII oocytes were parthenogenetically activated with strontium chloride. The development of the *Lin28a*-injected oocytes to the PN and 2C stages (17.7% and 15.7%, respectively) was similar to that of non-injected oocytes (20.8% and 21.6%) and of control siRNA-injected oocytes (17.2% and 16.4%; figure 5*a*). The role of Lin28a in preparing the oocyte for fertilization was further evaluated by *in vitro* fertilization (IVF). We found that PN formation was similar in the three groups (non-injected, 74.1%; control siRNA, 71.2%; *Lin28a* siRNA, 74.2%; figure 5*b*).

#### 3.5.2. *Lin28a* silencing causes embryonic arrest at the two-cell stage

**Figure 6.**
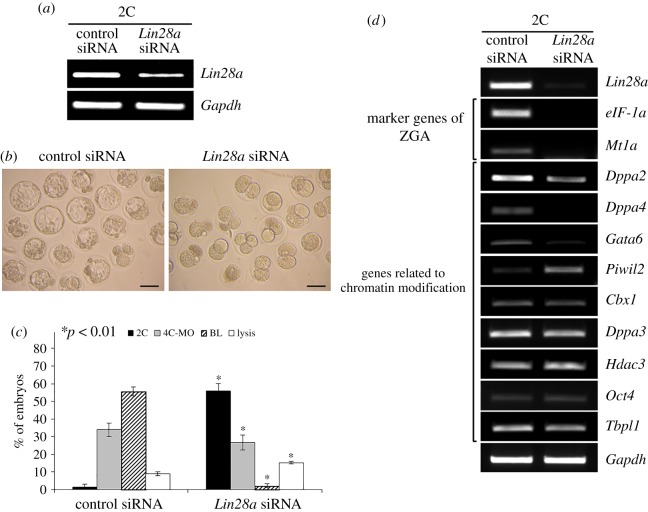
*Lin28a* siRNA caused developmental arrest at the 2C stage. (*a*) Microinjections of *Lin28a* siRNA reduced *Lin28a* mRNA levels, as assessed by RT-PCR. (*b*) Images of embryos 3 days after RNAi delivery. Most of the *Lin28a* siRNA-injected PN embryos were arrested at the 2C stage, whereas most of the control embryos developed further, up to the BL stage. Scale bars, 100 µm. (*c*) The developmental stage of PN embryos microinjected with control or *Lin28a* siRNA was scored after 3 days. The results are expressed as the mean ± s.e.m. of three experiments. *p < 0.01 compared with control siRNA-injected embryos (same stage). (*d*) Effect of *Lin28a* siRNA on the expression of genes related to ZGA and chromatin modification in embryos. PN embryos injected with control siRNA or *Lin28a* siRNA were matured *in vitro*. 2C-stage embryos were used to analyse gene expression.

Because *Lin28a* RNAi did not affect oocyte maturation, activation, fertilization or PN formation, we evaluated the effect of *Lin28a* RNAi on preimplantation embryonic development. *Lin28a* RNAi reduced *Lin28a* mRNA levels by 80% compared with control siRNA (figure 6*a*) and blocked embryo development (figure 6*b*). Most of the control siRNA-injected PN embryos developed into four-cell MOs (33.92%) and BLs (55.66%), and only 1.52% of these embryos remained at the 2C stage (figure 6*c*). By contrast, the *Lin28a*-silenced embryos arrested at the 2C stage (55.82%); only 2.19% of these embryos developed to the BL stage (figure 6*c*). We found that ZGA markers (*eIF-1a* and *Mt1a*) were dramatically downregulated in *Lin28a*-silenced 2C stage embryos (figure 6*d*). Some chromatin modification genes were also downregulated (*Dppa2*, *Dppa4* and *Gata6*), whereas *Piwil2* was upregulated. By contrast, the expression of other chromatin modification genes (*Cbx1*, *Dppa3*, *Hdac3*, *Oct4* and *Tbpl1*) did not change significantly (figure 6*d*).

### 3.6. The miR-125 family suppresses early embryonic development via regulation of zygotic genome activation

**Figure 7.**
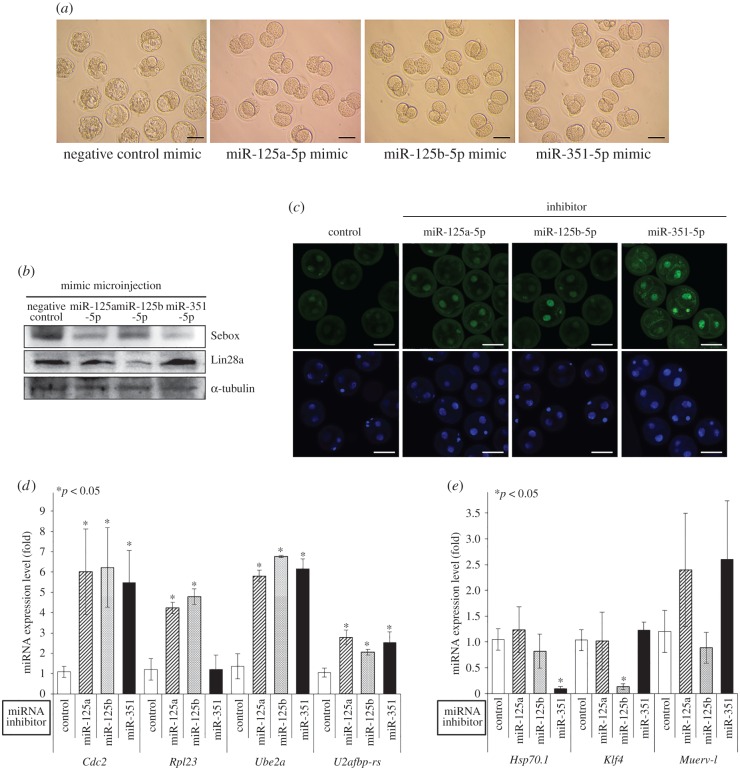
Simultaneous suppression of Sebox and Lin28a through treatment with mimics of miR-125 family members impaired early embryogenesis, resulting in the arrest of embryogenesis at the 2C stage. (*a*) Treatment with mimics of each miR-125 family member resulted in the arrest of embryo development at the 2C stage. Scale bars, 100 µm. (*b*) The treatment with miR-125 family member mimics reduced Sebox and/or Lin28a protein levels. Western blot results of endogenous Sebox and Lin28a expression in arrested 2C embryos that were microinjected with a negative control mimic or with mimics of each miR-125 family member. The blots were reprobed with an α-tubulin antibody as a loading control. (*c*) Embryonic transcriptional activity assay of miR-125 family-silenced 2C embryos. After inhibitors of the miR-125 family were microinjected into PN embryos, transcriptional activity was investigated based on nuclear EU incorporation (green) in 2C embryos. DNA was counterstained with Hoechst 33342 (blue). Scale bars, 50 µm. (*d*,*e*) Expression of ZGA markers in miR-125 family-depleted 2C embryos. The transcript level of ZGA markers was measured via quantitative real-time RT-PCR after PN embryos were treated with inhibitors of each miR-125 family member. *p < 0.05 compared with the control group.

We further investigated the effect of the miR-125 family on early embryonic development. As expected, microinjection of miR-125 family member mimics significantly affected embryonic development, causing it to stop at the 2C stage; the negative control mimic did not have this effect (figure 7*a* and table 4). The majority of the miR-125a-5p mimic-injected (84.3%), miR-125b-5p mimic-injected (65.5%) and miR-351-5p mimic-injected PN embryos (71.2%) were arrested at the 2C stage (table 4), similar to the effects of directly targeting *Sebox* [17] and *Lin28a* by dsRNA microinjection (figure 6). In addition, Sebox and Lin28a translation was markedly decreased by the overexpression of each miR-125 family member mimic in 2C embryos, except that the miR-351-5p mimic did not affect Lin28a translation (figure 7*b*). Taken together, these results strongly suggest that microinjection of miR-125 family member mimics caused abnormally low levels of Sebox and Lin28a in PN embryos, which led to developmental arrest of the early stage embryos.

**Table 4.** *In vitro* development of mouse GV oocytes after PN embryos were microinjected with mimics of each miR-125 family member.

	number of embryos (%)					
	total	2 cell	3 cell	4/8 cell	MO	BL
negative control mimic	57	0 (0.0)	0 (0.0)	10 (16.6)	9 (17.0)	38 (66.4)
miR-125a-5p mimic	40	34 (84.2)^a^	3 (7.9)	3 (7.9)	0 (0.0)	0 (0.0)^a^
miR-125b-5p mimic	31	17 (65.5)^a^	12 (29.7)	1 (2.2)	1 (2.6)	0 (0.0)^a^
miR-351-5p mimic	42	29 (71.2)^a^	9 (21.2)	4 (7.6)	0 (0.0)	0 (0.0)^a^

^a^Values are statistically significant at *p*<0.001 compared with the negative control mimic-injected group at the same stage.

To determine whether downregulation of the miR-125 family contributes to ZGA, we evaluated transcriptional activity in 2C embryos microinjected with inhibitors of the miR-125 family through EU incorporation. After the microinjection of each miR-125 family inhibitor, 2C embryos showed increased EU incorporation compared with the control group (figure 7*c*). These results indicate that the downregulation of the miR-125 family leads to increased transcriptional activity via upregulation of *Sebox* and *Lin28a*. In addition, we determined the expression of several ZGA markers in 2C embryos treated with inhibitors of the miR-125 family. As shown in figure 7*d*, the expression levels of *Cdc2*, *Rpl23*, *Ube2a* and *U2afbp-rs* were significantly upregulated, whereas no change in *Rpl23* mRNA expression was observed under treatment with the inhibitor of miR-351-5p. By contrast, when PN embryos were microinjected with the inhibitors of miR-125b-5p and miR-351-5p, there was a marked reduction of the transcript levels of *Klf4* and *Hsp70.1*, respectively, but no change in the expression of the remaining mRNAs was observed (figure 7*e*). The expression of *Muerv-l* was not significantly altered (figure 7*e*). These results strongly suggest that the miR-125 family is involved in the development of embryos beyond the 2C stage, through regulation of ZGA via control of *Sebox* and *Lin28a* expression.

### 3.7. Bioinformatics results suggest that the miR-125 family targets most maternal effect genes related to two-cell arrest

**Figure 8.**
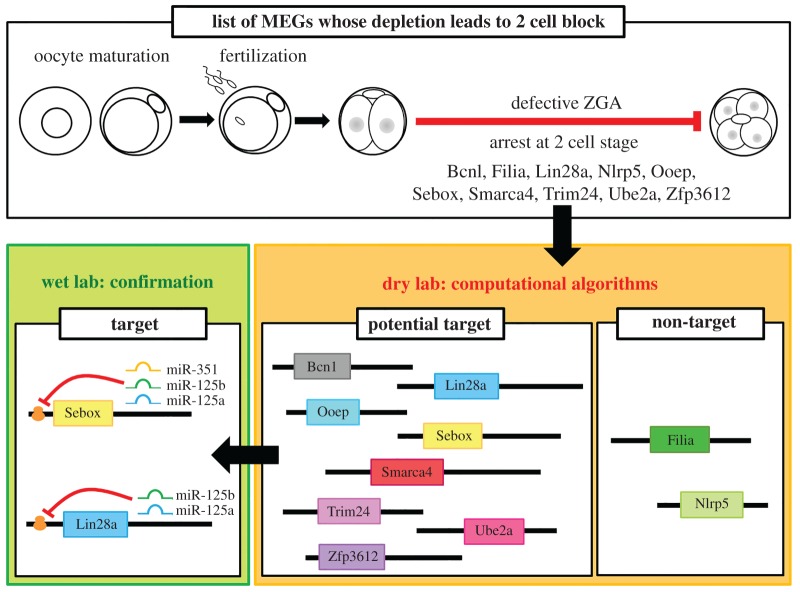
Schematic diagram depicting the identification of potential target genes of the miR-125 family using computational algorithms. The depletion of MEGs, such as *Bcn1*, *Filia*, *Lin28a*, *Nlrp5*, *Ooep*, *Sebox*, *Smarca4*, *Trim24*, *Ube2a* and *Zfp36l2*, has been reported to result in defective embryogenesis, especially arrest at the 2C stage due to impaired ZGA. Using computational algorithms, we found that all of these MEGs, excluding *Filia* and *Nlrp5*, contained a binding motif for the seed region of the miR-125 family in the 3′UTR of their mRNA. Using wet laboratory experimental methods, we found that of these MEGs, Sebox and Lin28a play an important role during ZGA and are direct targets of miR-125 family members in oocytes and embryos. However, further wet laboratory experimental studies are needed to confirming the data acquired through dry laboratory methods.

The MEGs accumulate maternal factors during oogenesis and enable ZGA and the progression of early embryo development [10]. The disruption of several MEGs did not affect folliculogenesis, oogenesis, oocyte maturation, ovulation or fertilization but did affect ZGA in 2C-stage embryos and subsequently led to arrest at the 2C stage [10]. Because we found that miR-125 family members concurrently suppressed Sebox and Lin28a, we searched for more potential MEG targets of the miR-125 family using computational algorithms. We focused on MEGs for which disrupted expression had been reported to result in 2C arrest. The miRNA target prediction process, or so-called dry laboratory method, identified more MEG genes (*Bcn1*, *Lin28a*, *Ooep*, *Sebox*, *Smarca4*, *Trim24*, *Ube2a* and *Zfp36l2*) as potential targets of the miR-125 family; *Filia* and *Nlrp5* were not identified as potential targets (figure 8; electronic supplementary material, table S2). Among the MEG genes identified as potential targets of the miR-125 family, we confirmed through wet laboratory methods that Sebox translation was suppressed by all members of the miR-125 family, while Lin28a translation was inhibited by miR-125a and miR-125b (figures 3, 7 and 8). Taking these results together, we suggest that most of the MEGs whose disruption resulted in 2C arrest were regulated by the miR-125 family. Further studies are required to assess whether the miR-125 family regulates ZGA by targeting the other predicted MEGs (*Bcn1*, *Ooep*, *Smarca4*, *Trim24*, *Ube2a* and *Zfp36l2*) in oocytes and embryos.

## 4. Discussion

miRNAs are endogenous non-coding RNAs that control gene expression at the transcriptional as well as translational level. Recently, studies on miRNAs have focused on *in silico* analyses using bioinformatics tools to identify target mRNAs and determine the regulatory mechanisms between miRNAs and proteins. Numerous computational approaches for miRNA target prediction have already been developed, such as TargetScan, miRanda, miRmap, Diana-MicroT, TargetBoost, miTarget, MirTarget2, TargetSpy, TargetMiner, MultiMiTar, NBmiRTar and microT-ANN [24]. Because each algorithm has its own set of limitations, multiple computational algorithms are commonly used simultaneously to predict and identify miRNA targets through comparisons [25]. For most algorithms, including PicTar [26], TargetScan [27], miRanda [28], miRmap [29], TargetMiner [30], PITA [31] and SVMicrO [32], target prediction is based on seed matching, conservation, free energy and site accessibility [33]. miRNA binding sites can be categorized into four main categories according to the Watson–Crick match (A-U and G-C) between an miRNA seed sequence and its target: (i) the 6mer is an exact match between the miRNA seed sequence and the target mRNA for six nucleotides; (ii) the 7mer-m8 is an exact match to positions 2–8 of the 5′ end of the miRNA, which contains the seed region; (iii) the 7mer-1A is an exact match to positions 1–7 of the 5′ end of the miRNA, which contains the seed region; and (iv) the 8mer is an exact match to positions 1–8 of the 5′ end of the miRNA, which contains the seed region [3,33].

A combination of computational algorithms was used for miRNA–mRNA target prediction. In this study, we employed the TargetScan [27], miRanda [28] and miRmap [29] algorithms to predict miRNA–mRNA interactions. Using this approach, we found that the miR-125 family targets the 3′UTRs of the *Sebox* and *Lin28a* mRNAs and that the miR-125 family members miR-125a, miR-125b and miR-351 share the same seed sequence [34]. Through computational searches, we found that these three closely related miRNAs in the miR-125 family bind to eigtht conserved base pairs within the 3′UTR sequence of *Sebox* and *Lin28a* mRNA, CUCAGGGA. According to the miRNA categories of TargetScan, these identified miRNAs were categorized into the 8mer category. The seed regions of the miR-125 family members and the MRE in the 3′UTR of *Sebox* and *Lin28a* were completely complementary.

The miR-125 family is highly conserved and is expressed in mammals [35]. As one of the most important miRNA families, the miR-125 family has been implicated in a variety of developmental processes, including host immune responses and cancer and disease development, as either a repressor or promoter [35]. Each member of the miR-125 family is located on different murine chromosomes and is most probably expressed in clusters with other miRNAs, such as miR-99/miR-100 and let-7 family members, suggesting that their expression is regulated by different 5′ UTR regulatory sequences and transcription factors [36]. Among the members of the miR-125 family, miR-125a and miR-125b have been shown to suppress *Lin28a* expression during the early stages of ESC differentiation [37]. In this study, we also observed miR-125a- and miR-125b-mediated post-transcriptional control of Lin28a in mESCs. Thus, we suggest that the expression of Lin28a in mESCs is strictly regulated by miR-125a and miR-125b. In addition, Lin28a binds with various miRNAs, such as let-7, miR-30 and miR-9, and has multiple roles in development, differentiation, growth, metabolism and pluripotency [37,38]. Further studies examining the finely tuned Lin28a-mediated regulation of embryonic development are needed.

Importantly, let-7e and miR-125a share the same genomic locus, suggesting that these miRNAs originate from the same primary miRNA transcript and regulate *Lin28a* expression [37]. These findings also suggest that *Lin28a* expression may be extensively and complexly controlled by miR-125 family members and other *Lin28a* regulatory miRNAs. Importantly, we showed that miR-351-5p, another member of the miR-125 family, did not significantly affect endogenous Lin28a translation in mESCs and oocytes. These data indicate that although members of the miR-125 family share the same seed region sequence, expression of the *Lin28a* target gene was regulated differently by each miR-125 family member.

*Sebox* is a relatively new MEG that encodes a subfamily of homeodomain-containing homeobox proteins. Most homeodomain-containing proteins act as transcription factors and activate or repress the expression of other target genes. Whether *Sebox* is a transcription factor that binds to and controls the activity of other genes remains unclear. Therefore, further studies should determine whether *Sebox* represents a novel transcription factor and clarify mutual functional interactions between *Sebox* and its target genes. In mice, *Sebox* has recently been shown to affect both early oogenesis in the fetus and early embryogenesis, and it has also been reported to affect mesoderm formation in early amphibian embryos [17,19,39,40]. In this study, we provide the first report that Sebox, both mRNA and protein, is expressed in mESCs. Until now, however, *Sebox* has not been specifically referenced in any stem cell research study. Our observations demonstrate that miR-125 family members negatively regulate Sebox translational levels in mESCs and oocytes in a seed sequence-dependent manner. Furthermore, luciferase reporter analyses revealed the direct binding of miR-125 family members to the 3′ UTR of *Sebox* mRNA. The findings of this study suggest that future studies of the regulatory mechanisms by which the miR-125 family and its target *Sebox* mRNA play a role in maintaining the pluripotency and differentiation of mESCs will be of considerable interest.

*Lin28a* is a conserved RNA binding protein that was originally identified as a key regulator of developmental timing in *Caenorhabditis elegans* [41]. *Lin28a* is abundant in human and mouse ESCs, but its expression decreases during differentiation [42]. *Lin28a* appears to suppress miRNA-mediated differentiation in stem cells [43]. In addition, *Lin28a* was used together with *Oct4*, *Sox2* and *Nanog* as a pluripotency reprogramming factor to reprogram fibroblasts to generate induced pluripotent stem cells [44]. Importantly, *Lin28a* is also highly expressed in oocytes and mESCs. Although *Lin28a* RNAi resulted in a marked decrease in both Lin28a mRNA and protein, these changes in oocytes did not affect the oocytes' competence for maturation and fertilization. Conversely, Lin28a knockdown in PN-stage embryos blocked preimplantational embryo development mainly at the 2C or 4C stage. Based on these observations of the present study, we conclude that *Lin28a* is a new MEG that has a pivotal function in early embryonic development, especially in 2C block and ZGA.

Maternal mRNAs and proteins accumulate in developing oocytes but are mostly degraded by the end of the 2C stage [45]. Maternal mRNAs and proteins are replaced by embryonic gene products during early embryonic development. This process, known as ZGA, is the first major developmental event that takes place after fertilization [46]. ZGA occurs around the PN to 2C stage in mice [47] and around the 4C to 8C stage in humans [48]. Zygotic gene activation is responsible for reprogramming gene expression, which establishes the totipotent state of each blastomere in cleavage-stage embryos [13]. *Sebox* plays a crucial role in preparing oocytes for embryonic development by orchestrating the expression of other important MEGs and ZGA markers [19]. Furthermore, our findings demonstrate that ZGA is also disrupted by *Lin28a* depletion. Therefore, we conclude that *Sebox* and *Lin28a* are mainly involved in the decay of maternal transcripts and ZGA during early embryogenesis. Likewise, as expected, we found that knockdown of the miR-125 family led to an increase Sebox and Lin28a protein levels during oocyte maturation and to an increase in the expression of genes related to ZGA during the MZT period. Based on these findings, we conclude that the miR-125 family is a novel regulator of ZGA that functions through the regulation of MEGs, for example, by modulating *Sebox* and *Lin28a* translation.

By using dry laboratory tools, we also found that miR-125 family members may regulate the expression of other MEGs that are involved in the 2C block of preimplantational embryonic development *in vitro*. This computational work has been inspired by the observation that miR-125 family members simultaneously regulate Sebox and Lin28a and that Sebox and Lin28a both function as MEGs during early embryonic development *in vitro*. As we expected, most of the MEGs related to features of the 2C block, excluding *Nlrp5* and *Filia*, seemed to be regulated by miR-125 family members. This observation is also a very interesting and meaningful discovery and may open a new area of research to study the regulatory mechanisms of the timely expression and degradation of MEGs during early embryonic development.

## 5. Conclusion

This is the first report on the pivotal role of miR-125 family members, miR-125a-5p, miR-125b-5p and/or miR-351-5p, in the regulation of MEG expression, especially the expression of *Sebox* and *Lin28a* in oocytes, embryos and mESCs. This regulation is controlled via direct binding of miR-125 family members to *Sebox* and *Lin28a* mRNA, which causes translational repression. Our findings provide a promising foundation for future studies of the regulation of MEGs and/or reprogramming factors by miRNAs in oocytes, embryos and mESCs.

## Supplementary Material

The miR-125 family is an important regulator of the expression and maintenance of maternal effect genes during preimplantational embryo development - Lee et al

## Data accessibility

The relevant data supporting this article have been uploaded as part of the electronic supplementary material, figures S1 and S2 and tables S1 and S2.
