# Supplementary material for: The miR-125 family is an important regulator of the expression and maintenance of maternal effect genes during preimplantational embryo development
Source: Open Biol. 2016 Nov 30;6(11):160181. doi: 10.1098/rsob.160181 (PMC5133438; doi:10.1098/rsob.160181)
Supplement: The miR-125 family is an important regulator of the expression and maintenance of maternal effect genes during preimplantational embryo development - Lee et al [file rsob160181supp1.pdf]

Supplementary materials

The miR-125 family is an important regulator of the expression and maintenance of maternal effect genes during preimplantational embryo development

Kyeoung-Hwa Kim[1], You-Mi Seo[2], Eun-Young Kim[1], Su-Yeon Lee[1],

Jini Kwon[1], Jung-Jae Ko[1,] * and Kyung-Ah Lee[1,] *

[1]Institute of Reproductive Medicine, Department of Biomedical Science, College of Life Science, CHA University, Pangyo, Korea. [2]Department of Oral Histology-Developmental Biology, School of Dentistry and Dental Research Institute, Seoul National University, Seoul, Korea.

Contact Information

*Correspondence and requests for materials should be addressed to J.J.K. (email: highko@cha.ac.kr) or K.A.L. (email: leeka@cha.ac.kr).

Running head: miR-125 family regulates MEG expression

Keywords: Sebox, Lin28a, miR-125 family, Translational regulation, Bioinformatics

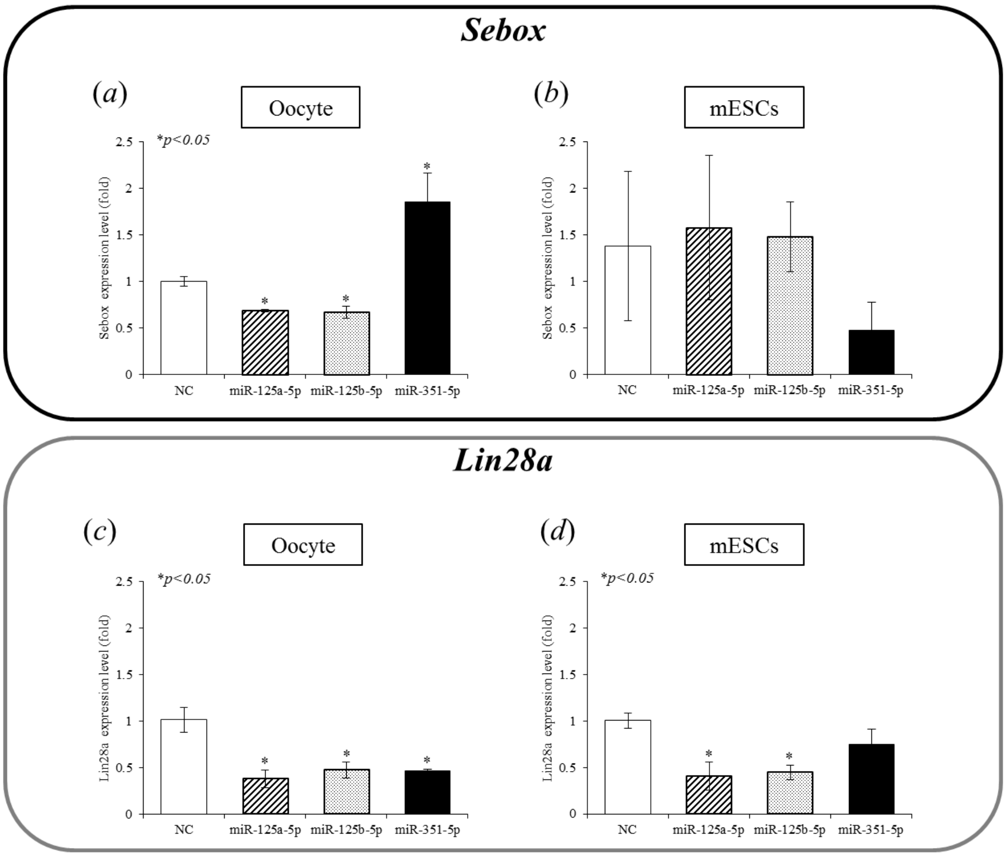

Supplementary Figure 1. Endogenous *Sebox* and *Lin28a* transcript levels were differentially regulated by different mimics in different cell types, including oocytes and mESC. The endogenous *Sebox* transcript level was measured by quantitative real-time RT-PCR after oocytes (*a*) and mESCs (*b*) were treated with mimics of each miR-125 family member. The endogenous *Lin28a* transcript level was measured after oocytes (*c*) and mESCs (*d*) were treated with mimics of each miR-125 family member. NC, treatment with negative control mimic. *$p<0.05$ compared with the negative control mimic-transfected or negative control mimic-microinjected group.

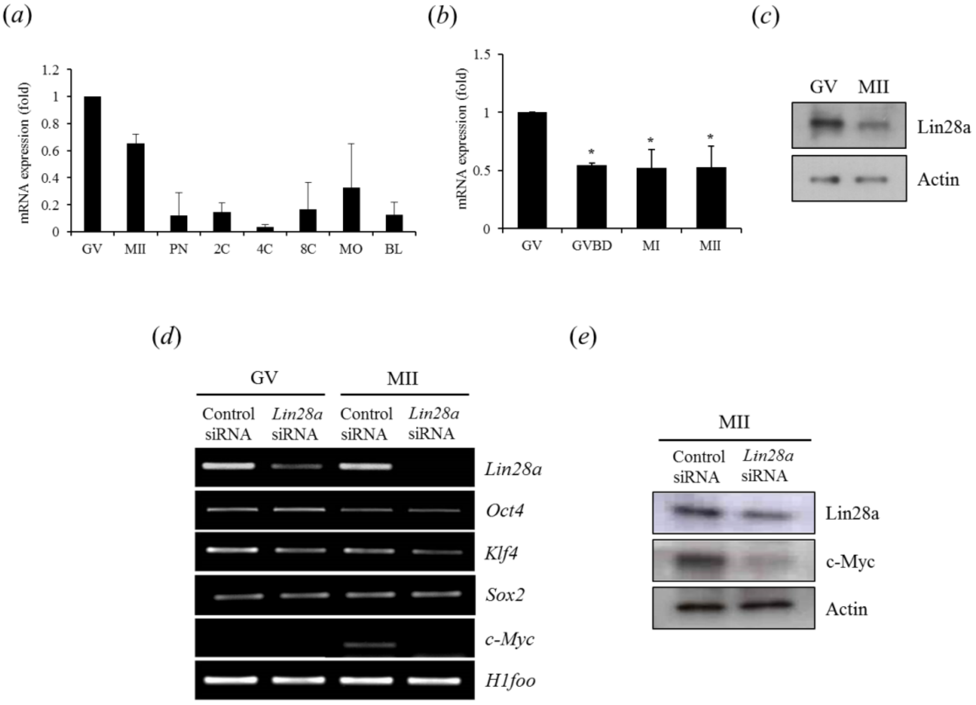

Supplementary Figure 2. Downregulation of c-Myc by *Lin28a* siRNA. (*a*) The expression pattern of *Lin28a* during early embryogenesis. The relative gene expression of *Lin28a* in a single oocyte or embryo throughout development was determined by quantitative real-time RT-PCR. Synthetic GFP RNA was used as an internal control for normalization. The results are expressed as the mean±SEM of at least three experiments. GV, germinal vesicle; MII, metaphase II; PN, pronuclear stage; 2C, 2-cell stage; 4C, 4-cell stage; MO, morula stage; BL, blastocyst stage. (*b*) Expression of *Lin28a* transcript in mouse oocytes. *Lin28a* mRNA levels during oocyte maturation were determined by quantitative real-time RT-PCR. *p<0.05 compared with GV oocytes. (*c*) Expression of *Lin28a* protein in mouse oocytes, as determined by Western blot analysis. The experiment was conducted three times, with 100 oocytes per lane. The blots were reprobed with an Actin antibody as a loading control. (*d*) *Lin28a* RNAi suppressed *Lin28a* and *c-Myc* mRNA levels but did not affect the expression of other reprogramming factors (*Oct4*, *Sox2*, and *Klf4*) in *Lin28a*-silenced oocytes. *H1foo* was used as an internal control. (*e*) *Lin28a* RNAi disrupts Lin28a and c-Myc protein expression, as confirmed by Western blot analysis. The lysates of 200 MII oocytes injected with control siRNA or *Lin28a* siRNA were separated by SDS-PAGE. Proteins were visualized using specific antibodies against Lin28a, c-Myc and Actin (loading control).

Supplementary Table 1. miR-125 family prediction score for Sebox and Lin28a using each computational algorithm.

		Algorithm		
		TargetScan	miRanda	miRmap
Gene	miRNAs	Context+ score[a]	mirSVRscore[b]	miRmapscore[c]
Sebox	miR-125a-5p	-0.37	-0.1229	84.41
	miR-125b-5p	-0.35	-0.1205	88.10
	miR-351-5p	-0.33	-0.1340	84.85
Lin28a	miR-125a-5p	-0.28	-0.6884	89.78
	miR-125b-5p	-0.28	-0.6847	89.30
	miR-351-5p	-0.29	-0.6847	91.25

The more negative the value of the context+ score[a] from TargetScan and the mirSVRscore[b] from miRanda, the more favorable and higher the probability of mRNA binding.

[c]The miRmap score ranges from 0 to 100, where 0 represents no complementarity and 100 represents complete complementary.

Supplementary Table 2. List of the MEGs identified as predicted targets of the miR-125 family via dry lab methods (i.e., using three computational algorithms: miRanda, TargetScan, and miRmap). Among the list of MEGs whose depletion has been reported to lead to embryonic arrest at the 2C stage, only *Filia* and *Nlrp5* appear to be unlikely targets of miR-125 family members; the rest of the MEGs were predicted as tentative targets of at least one of the miR-125 family members.

	Computational algorithm		
Gene	miRanda	TargetScan	miRmap
Filia	-	-	-
Nlrp5	-	-	-
Bnc1	-	miR-125b-3p	miR-125b-3p
Lin28a	miR-125a-5p<br>miR-125b-5p<br>miR-351	miR-125a-5p<br>miR-125b-5p<br>miR-125b-3p<br>miR-351-5p	miR-125a-5p<br>miR-125b-5p<br>miR-351-5p
Ooep	-	miR-125a-3p	miR-125a-3p
Sebox	miR-125a-5p<br>miR-125b-5p<br>miR-351	miR-125a-5p<br>miR-125b-5p<br>miR-351-5p	miR-125a-5p<br>miR-125b-3p<br>miR-125b-5p<br>miR-351-5p
Smarca4	-	miR-125a-3p<br>miR-125b-3p	miR-125b-3p
Trim24	-	miR-125a-5p<br>miR-125a-3p<br>miR-125b-5p<br>miR-351-5p<br>miR-351-3p	-
Ube2a	miR-125a-5p<br>miR-125b-5p<br>miR-351	miR-351-3p	-
Zfp36l2	miR-125a-3p	miR-125a-3p	miR-125a-3p
